# Supplementary material for: Oxidative stress-induced FABP5 S-glutathionylation protects against acute lung injury by suppressing inflammation in macrophages
Source: Nat Commun. 2021 Dec 7;12:7094. doi: 10.1038/s41467-021-27428-9 (PMC8651733; doi:10.1038/s41467-021-27428-9)
Supplement: Supplementary file 1 — Supplementary Information [file 41467_2021_27428_MOESM1_ESM.pdf]

Supplementary Figures

Supplementary Fig 1

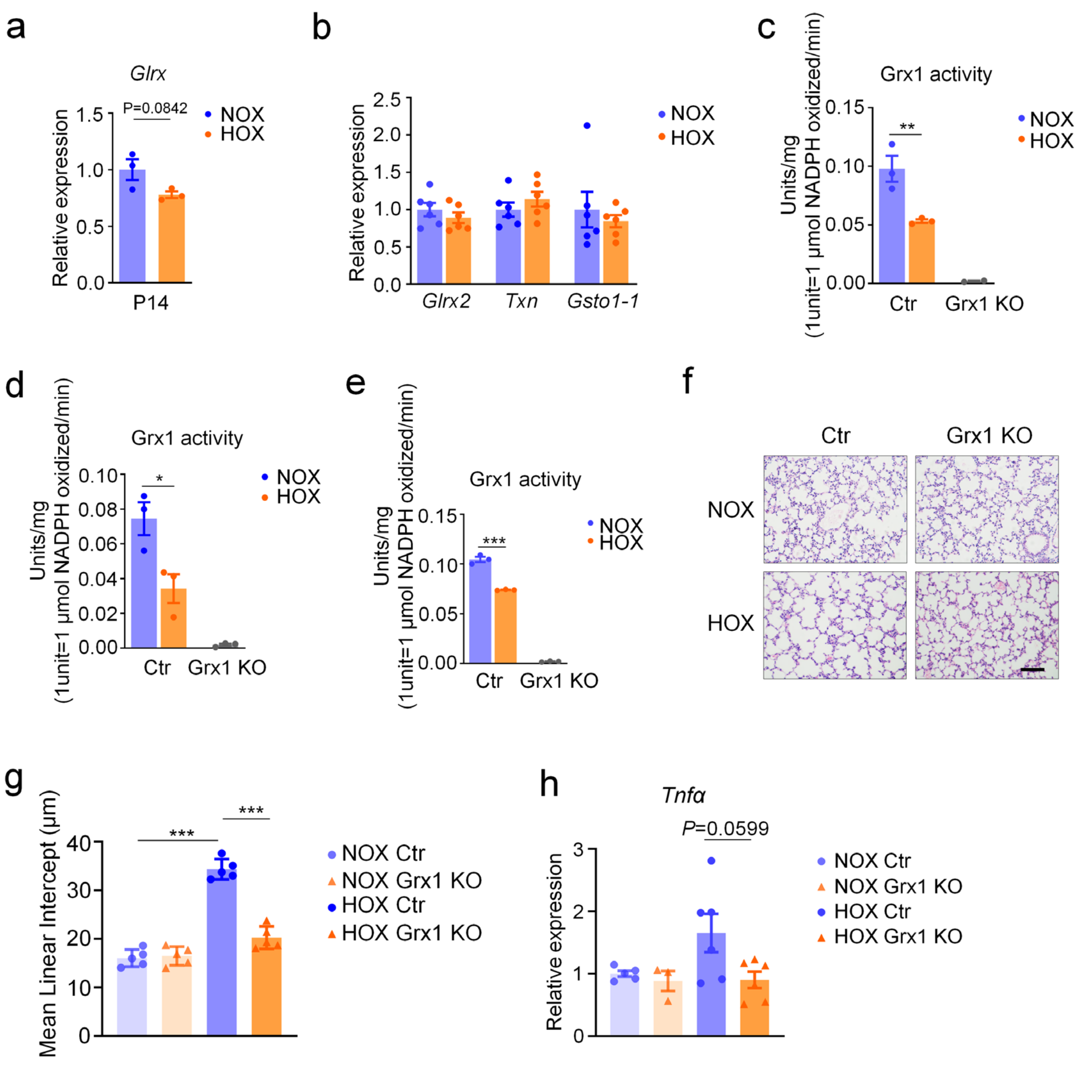

Supplementary Fig. 1 Grx1 KO mice show decreased inflammation in hyperoxia-induced acute lung injury.

a Grx1 mRNA expression in lung tissue harvested at P14 after exposure to NOX(n=3) or HOX(n=3), as determined by qPCR. **b** mRNA levels of enzymes (*Glrx2, Txn, Gsto1-1*) involved in protein S-glutathionylation in lung tissue harvested after exposure to NOX or HOX as evaluated by qPCR, n=6 in each group. **c** Grx1 activity (reaction mixtures containing 2-hydroxyethyl disulfide) in lung tissue at P7 after exposure to NOX or HOX. Lung tissue from Grx1 KO mice is a negative control, n=3 in each group, P = 0.0067. **d** Grx1 activity (reaction mixtures containing L-CySSG) in lung tissue at P14 after exposure to NOX or HOX. Lung tissue from Grx1 KO mice is a negative control, n=3 in each group, P =0.0184. **e** Grx1 activity (reaction mixtures containing 2-hydroxyethyl disulfide) in lung tissue at P14 after exposure to NOX or HOX. Lung tissue from Grx1 KO mice is a negative control, n=3 in each group, P <0.0001. **f**

Representative images of H&E staining of P14 control and Grx1 KO mice exposed to NOX or HOX (scale bars, 100 μm). **g** Morphometric analyses of lung by mean linear intercept (MLI) in all groups as in **f**, n=5 in each group, ****P* <0.0001. **h** mRNA levels *Tnfα* in lung tissue from control and Grx1 KO mice exposed to NOX or HOX as determined by qPCR, n=5, 3, 6, 6, respectively.
All samples were biologically independent and three or more independent experiments were performed. All quantitative data are shown as mean ± SEM and analyzed with a 95% confidence interval. Two-tailed unpaired Student's *t*-test for (a-b); one-way ANOVA followed by Tukey's post-hoc test for (c-e, g-h). * $P < 0.05$, ** $P < 0.01$, *** $P < 0.001$. Source data are provided as a Source Data file.

Supplementary Fig 2

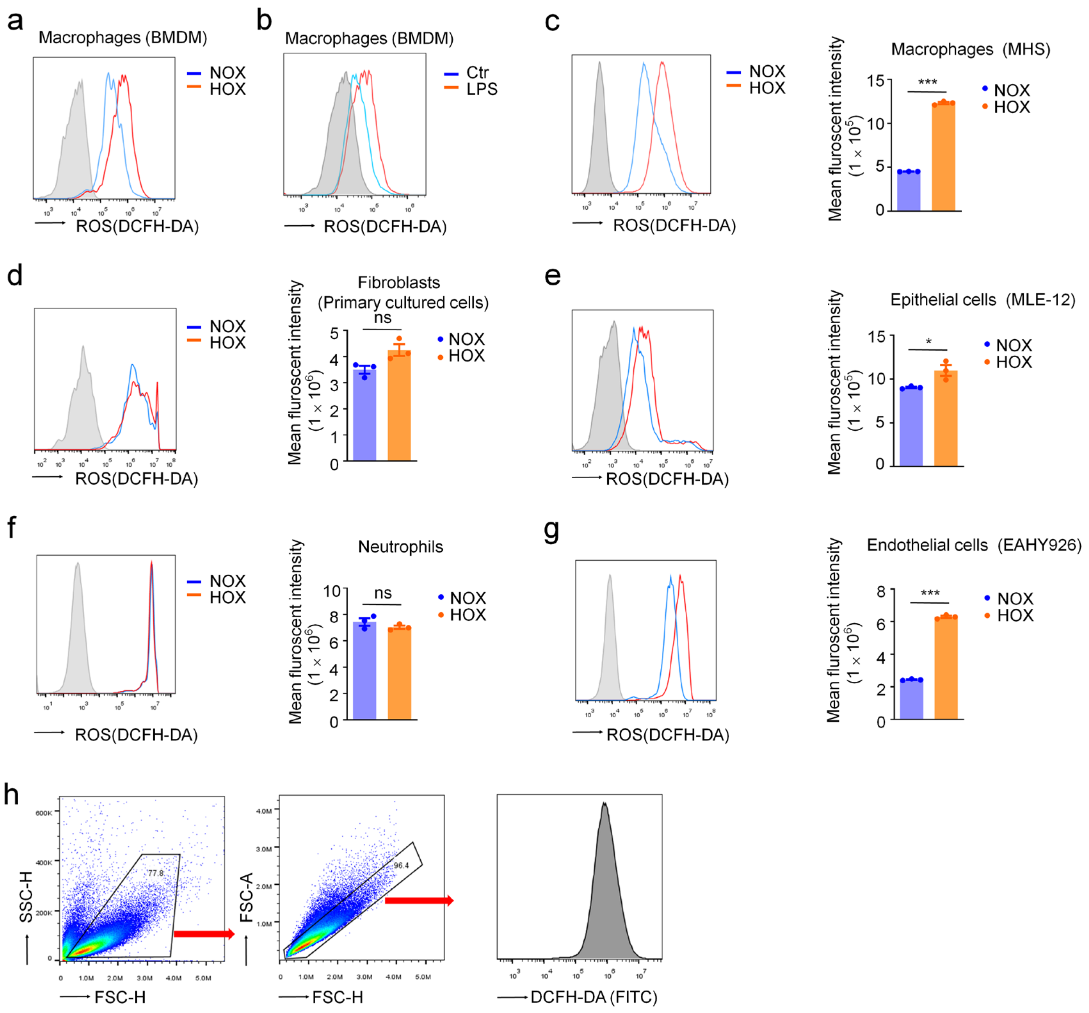

Supplementary Fig. 2 Intracellular ROS levels in different cells after exposure to hyperoxia.

a Intracellular ROS levels assayed with the DCFH-DA fluorescent probe in BMDMs cultured in NOX or HOX as determined by flow cytometry, n=3 in each group. **b** Representative plots of ROS generation in BMDMs stimulated with LPS (100 ng/mL) for 5 min as analyzed by flow cytometry, n=3 in each group. **c-g**, Representative plots and quantification of intracellular ROS levels assayed with the DCFH-DA fluorescent probe in MH-S (**c**), fibroblasts (**d**), MLE-12 (**e**), neutrophils (**f**), and EA.hy926 (**g**) cultured under NOX or HOX as determined by flow cytometry. **h** FACS gating strategy of intracellular ROS detection (**a-g**) by using MH-S as an example.

n=3 in each group, P_c<0.0001, P_d =0.0519, P_e =0.0361, P_f =0.2663, P_g <0.0001.

All samples were biologically independent and three or more independent experiments were performed. All quantitative data are shown as mean ± SEM and analyzed with a 95% confidence interval. Two-tailed unpaired Student's *t*-test for (c-g); * $P < 0.05$, ** $P < 0.01$, *** $P < 0.001$, ns=no significance. Source data are provided as a Source Data file.

Supplementary Fig 3

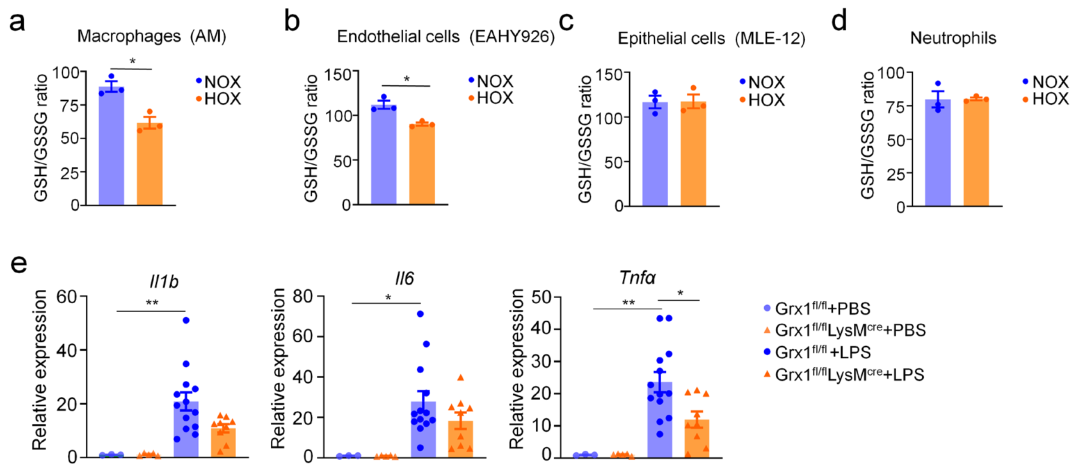

Supplementary Fig. 3 GSH/GSSG ratios in different cells after hyperoxia and the LPS-induced cytokine levels in lung tissue from Grx1$^{fl/fl}$ and Grx1$^{fl/fl}$LysM^{cre} mice. **a-d** GSH/GSSG ratios of AM (**a**), EA.hy926 (**b**), MLE-12 (**c**), and neutrophils (**d**) after culture under NOX or HOX, n=3 in each group, $P_a = 0.01$, $P_b = 0.0116$, $P_c= 0.9501$, $P_d = 0.9581$. **e** mRNA levels of *Il1b*, *Il6*, and *Tnfα* in lung tissue from Grx1$^{fl/fl}$ and Grx1$^{fl/fl}$LysM^{cre} mice 24 h following intratracheal administration of PBS or LPS (5 mg/kg) as evaluated by qPCR, n=3, 5, 13, 9, respectively, $P_{(Il1b,\ \mathrm{Grx1fl/fl\ +PBS\ vs.\ Grx1fl/fl\ +LPS})} = 0.0069$, $P_{(Il6,\ \mathrm{Grx1fl/fl\ +PBS\ vs.\ Grx1fl/fl\ +LPS})} = 0.0336$, $P_{(Tnf\alpha,\ \mathrm{Grx1fl/fl\ +PBS\ vs.\ Grx1fl/fl\ +LPS})} = 0.0023$, $P_{(Tnf\alpha,\ \mathrm{Grx1fl/fl\ +LPS\ vs.\ Grx1fl/fl\ LysMcre+LPS})} = 0.0246$.

All samples were biologically independent and three or more independent experiments were performed. All quantitative data are shown as mean ± SEM and analyzed with a 95% confidence interval. Two-tailed unpaired Student's *t*-test for (a-d); one-way ANOVA followed by Tukey's post-hoc test for (e). * $P < 0.05$, ** $P < 0.01$, *** $P < 0.001$. Source data are provided as a Source Data file.

Supplementary Fig 4

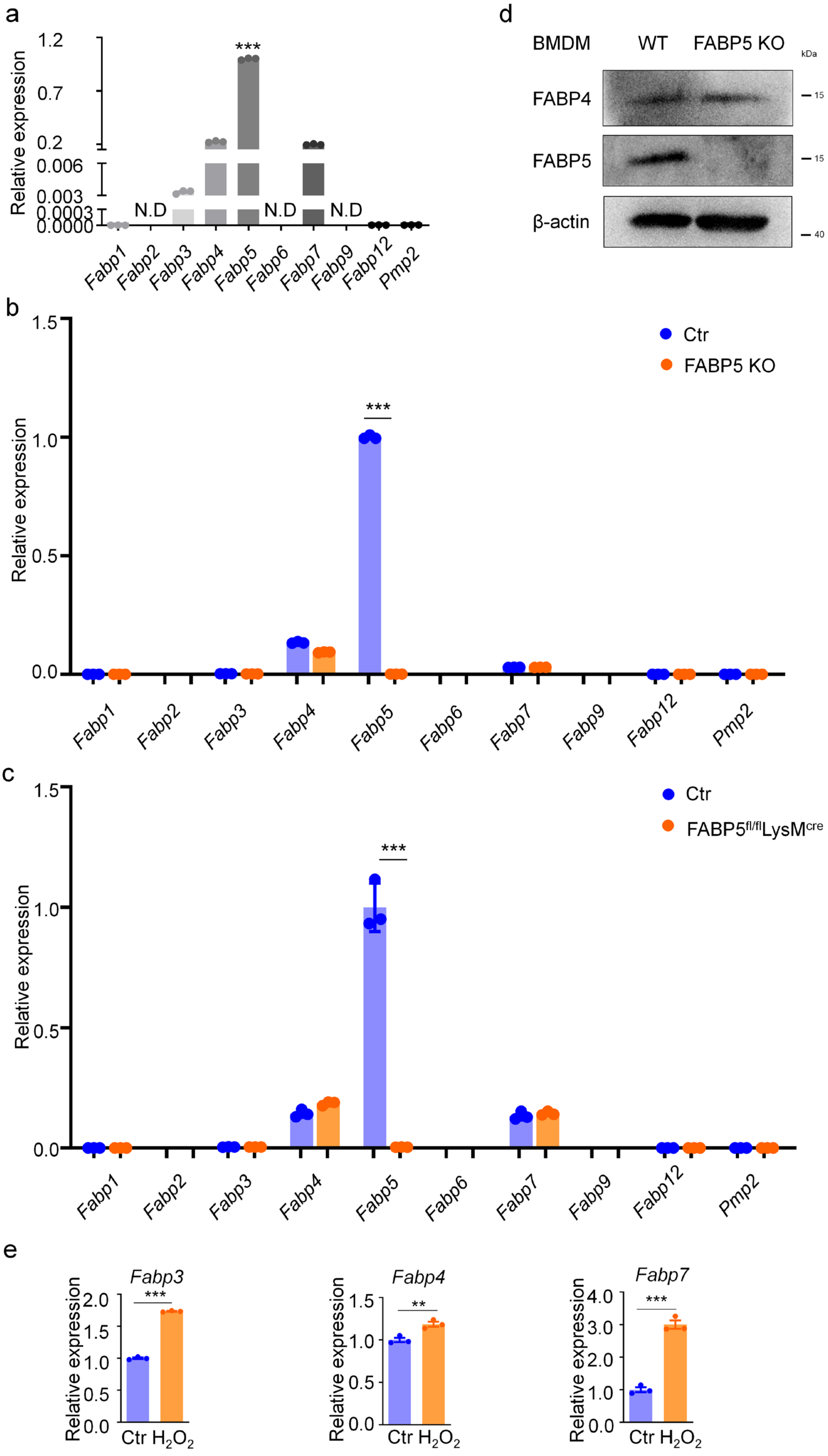

Supplementary Fig. 4 Expression levels of FABP family proteins under oxidative stress.

a mRNA levels of FABP family members in BMDMs determined by qPCR, n=3 in each group, P <0.0001. **b** mRNA levels of FABP family members in control and FABP5 KO BMDMs measured by qPCR, n=3 in each group, P <0.0001. **c** mRNA levels of FABP family members in BMDMs from FABP5$^{fl/fl}$ and FABP5$^{fl/fl}$LysM^{cre} mice determined by qPCR, n=3 in each group, P <0.0001. **d** levels of FABP4 and FABP5 evaluated by immunoblotting in control and FABP5 KO BMDMs. **e** mRNA levels of *Fabp3*, *Fabp4*, and *Fabp7* in RAW264.7 cells after exposure to H_2O_2 (500 μM), n=3 in each group, P_{Fabp3} <0.0001, P_{Fabp4} = 0.0093, P_{Fabp7} = 0.0002.

All samples were biologically independent and three or more independent experiments were performed. All quantitative data are shown as mean ± SEM and analyzed with a 95% confidence interval. Two-tailed unpaired Student's *t*-test for (e); one-way ANOVA followed by Tukey's post-hoc test for (a-c). * $P < 0.05$, ** $P < 0.01$, *** $P < 0.001$. Source data are provided as a Source Data file.

Supplementary Fig 5

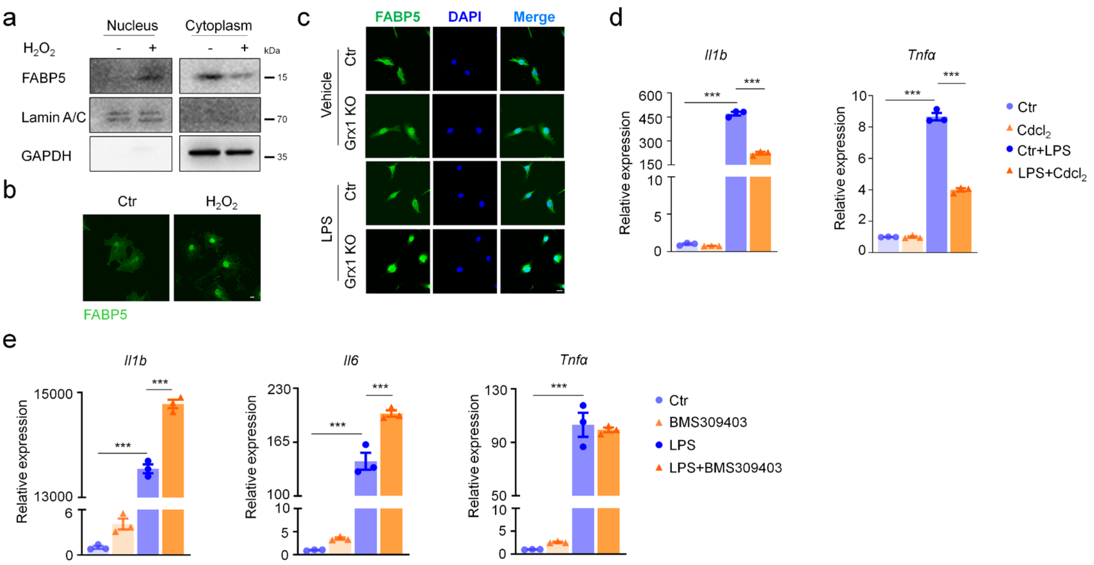

Supplementary Fig. 5 Cytokine levels in macrophages after treatment with Grx1 inhibitor or FABP5 inhibitor.

a Immunoblot analysis of cytoplasmic and nuclear FABP5 in COS-7 cells exposed to H_2O_2 (200 μM) for 1 h. Lamin A/C (nuclear fraction) and GAPDH (cytoplasmic fraction) are the loading controls. **b** Immunofluorescence staining (with FABP5) and confocal microscopy imaging of COS-7 cells after exposure to H_2O_2 (200 μM) for 1 h (green, FABP5; blue, DAPI; scale bars, 10 μm. **c** Immunofluorescence staining (with FABP5) and confocal microscopy imaging of control and Grx1 KO BMDMs after treatment with LPS (100 ng/mL) for 30 min (green, FABP5; blue, DAPI; scale bars, 10 μm). **d** mRNA expression of *Il1b* and *Tnfα* in RAW264.7 cells pre-treated with $CdCl_2$ (2 μM) for 30 min, then stimulated with LPS (100 ng/mL) for 4 h, as evaluated by qPCR, n=3 biologically independent samples over 3 independent experiments, data are presented as mean ± SEM and analyzed with a 95% confidence interval, ***P <0.0001, one-way ANOVA is followed by Tukey's post-hoc test. **e** mRNA expression of *Il1b*, *Il6*, and *Tnfα* in BMDMs pre-incubated with BMS309403 (50 μM) for 2 h, then stimulated with LPS (100 ng/mL) for 4 h, as determined by qPCR, n=3 biologically independent samples over 3 independent experiments, data are presented as mean ± SEM and analyzed with a 95% confidence interval, $P_{(Il16,\ \mathrm{LPS\ vs.\ LPS+\ BMS\ 309403})}$= 0.0003, ***$P$ <0.0001 (except group *Il16*, LPS vs. LPS+ BMS 309403), one-way ANOVA is followed by Tukey's post-hoc test. Source data are provided as a Source Data file.

Supplementary Fig 6

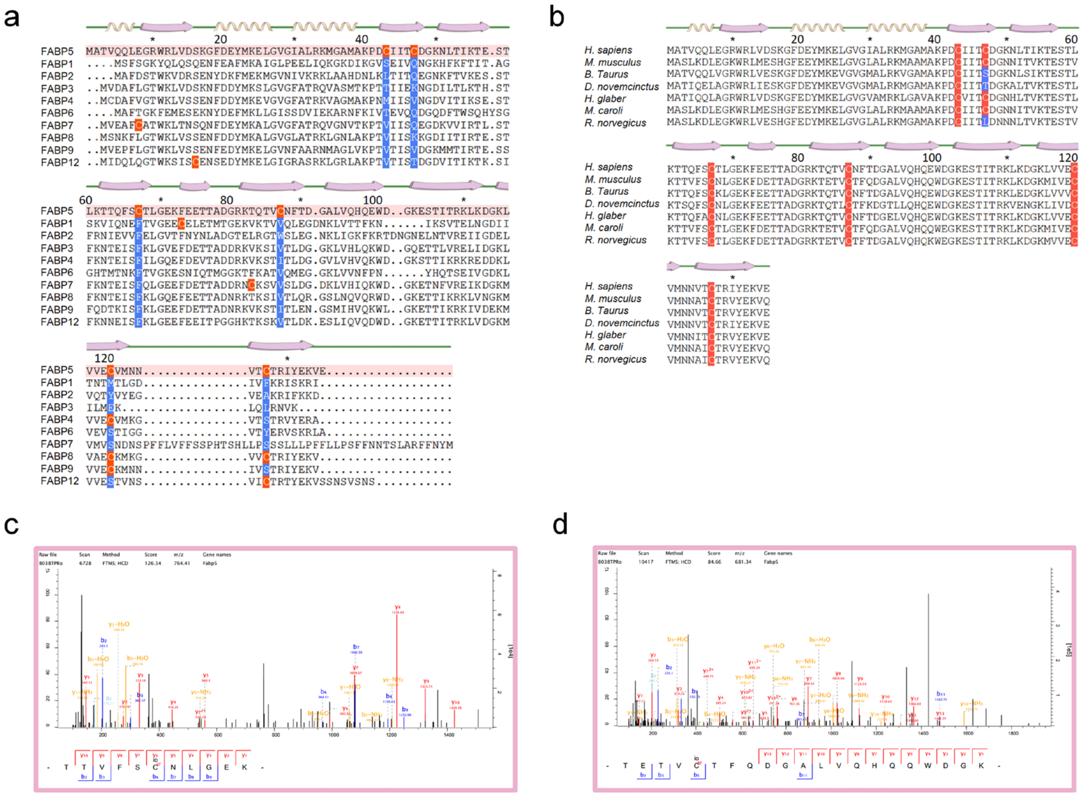

Supplementary Fig. 6 Determination of S-glutathionylation on Cys67 and 87 of FABP5 by LC-MS/MS.

a Sequence alignment around 6 cysteines of FABP5 with other members of the FABP family from *mus sapiens*. **b** Sequence alignment around 6 cysteines of FABP5 from different organisms. **c** Mass spectrum of a peptide from FABP5 including glutathionylated cysteine 67. **d** Mass spectrum of a peptide from FABP5 including glutathionylated cysteine 87.

Supplementary Fig 7

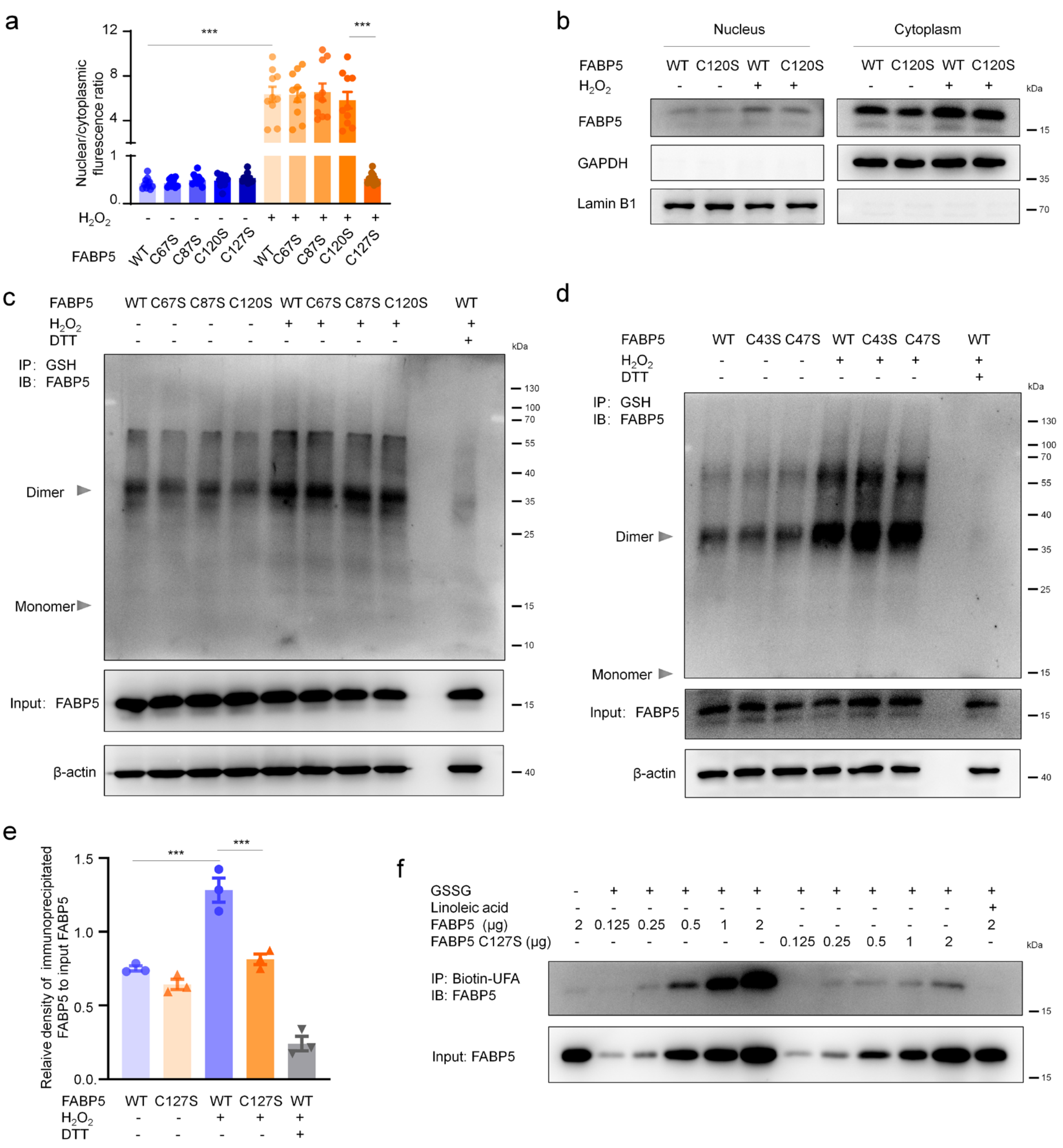

Supplementary Fig. 7 S-glutathionylation of Cys67, 87, 120 does not influence FABP5 nuclear translocation in response to ROS.

a Quantitative analysis of the ratio of nuclear/cytoplasmic fluorescence intensity in COS-7 cells as in Fig. 6e, n=10 biologically independent samples over 3 independent experiments, data are presented as mean ± SEM and analyzed with a 95% confidence interval, ****P* <0.0001, one-way ANOVA is followed by Tukey's post-hoc test. **b** Immunoblot analysis of cytoplasmic and nuclear FABP5 in COS-7 cells transfected with FABP5 WT or C120S after exposure to H_2O_2 (200 μM) for 1 h. Lamin B1 (nuclear fraction) and GAPDH (cytoplasmic fraction) are loading controls. **c** Co-IP for S-glutathionylation of FABP5 in COS-7 cells overexpressing pXJ40-3xFlag-FABP5 WT, C67S, C87S or C127S and exposed to H_2O_2 (200 μM) for 15 min (IP, GSH; IB, FABP5). Whole cell lysates confirm the expression of FABP5 and β-actin

(DTT, negative control). **d** Co-IP for S-glutathionylation of FABP5 in COS-7 cells overexpressing pXJ40-3xFlag-FABP5 WT, C43S or C47S and treated with H_2O_2 (200 μM) for 15 min (IP, GSH; IB, FABP5). Whole cell lysates confirm the expression of FABP5 and β-actin (DTT, negative control). **e** Relative expression of protein S-glutathionylation in COS-7 cells overexpressing pXJ40-3xFlag-FABP5 WT or C127S and exposed to H_2O_2 (200 μM) for 15 min (IP, GSH; IB, FABP5) as in Fig. 6j. Optical density is normalized by input FABP5, n=3 biologically independent samples over 3 independent experiments, data are presented as mean ± SEM and analyzed with a 95% confidence interval, $P_{(\text{FABP5 vs. FABP5+H2O2})}= 0.0001$, $P_{(\text{FABP5+H2O2 vs. FABP5 C127S+H2O2})}= 0.0004$, one-way ANOVA is followed by Tukey's post-hoc test. **f** Immunoblot analysis of FABP5 fatty acid binding. Different amounts of purified recombinant FABP5 WT or C127S protein was incubated with GSSG (1 mM) for 15 min, then mixed with Biotin-linoleic acid for 30 min. FABP5 that associated with the Biotin-linoleic acid was captured using streptavidin magnetic beads.

Source data are provided as a Source Data file.

Supplementary Fig 8

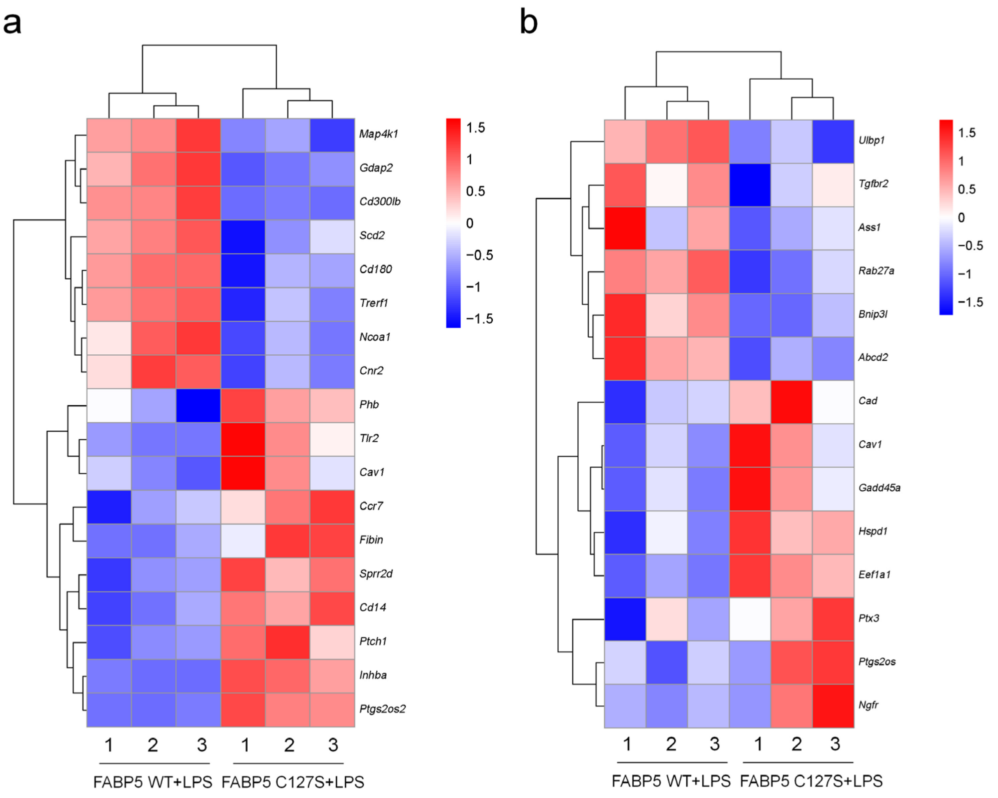

Supplementary Fig. 8 Heatmap of differentially-expressed genes associated with lipid response and ROS biosynthetic/metabolic process in LPS-stimulated BMDMs overexpressing FABP5 WT or C127S.

a Heatmap of response to lipid transcripts in LPS-stimulated (24 h) BMDMs nucleofected with either pXJ40-3xFlag-FABP5 WT or C127S. Data are relative to the calculated Z scores across samples (red, relatively high levels of expression; blue, relatively low levels of expression). Each column represents one individual (for a total of 3 per group) and each row represents the expression of a single gene. **b** Heatmap of ROS biosynthetic/metabolic process transcripts in LPS-stimulated (24 h) BMDMs nucleofected with either pXJ40-3xFlag-FABP5 WT or C127S. Data are relative to the calculated Z scores across samples (red, relatively high levels of expression; blue, relatively low levels of expression). Each column represents one individual (for a total of 3 per group) and each row represents the expression of a single gene.

Supplementary Table 1. Primers used in this study

Primers for qPCR		
Gene	Direction	Primer (5′–3′)
Actb	F (forward):	GGCTGTATTCCCCTCCATCG
	R (reverse):	CCAGTTGGTAACAATGCCATGT
Tnfα	F:	CCTGTAGCCCACGTCGTAG
	R:	GGGAGTAGACAAGGTACAACCC
Il1b	F:	GAAATGCCACCTTTTGACAGTG
	R:	TGGATGCTCTCATCAGGACAG
Il6	F:	CTGCAAGAGACTTCCATCCAG
	R:	AGTGGTATAGACAGGTCTGTTGG
Fabp5	F:	TGAAAGAGCTAGGAGTAGGACTG
	R:	CTCTCGGTTTTGACCGTGATG
Fabp4	F:	AAGGTGAAGAGCATCATAACCCT
	R:	TCACGCCTTTCATAACACATTCC
Fabp1	F:	ATGAACTTCTCCGGCAAGTACC
	R:	CTGACACCCCCTTGATGTCC
Fabp2	F:	GTGGAAAGTAGACCGGAACGA
	R:	CCATCCTGTGTGATTGTCAGTT
Fabp3	F:	ACCTGGAAGCTAGTGGACAG
	R:	TGATGGTAGTAGGCTTGGTCAT
Fabp6	F:	CTTCCAGGAGACGTGATTGAAA
	R:	CCTCCGAAGTCTGGTGATAGTTG
Fabp7	F:	GGACACAATGCACATTCAAGAAC
	R:	CCGAACCACAGACTTACAGTTT
Fabp9	F:	CACTGCAGACAACCGAAAAG
	R:	TCTGTTTGCCAAGCCATTTT
Fabp12	F:	TTGAAAACTACATGAAGGAATTGG
	R:	AGTGGGCTTTGCCAGACA
Pmp2	F:	AAGCTCTAGGTGTGGGGTTAG
	R:	AGTGTCACGATGCTCTTGGC
18s	F:	CGTCTGCCCTATCAACTTTCG
	R:	GCCTGCTGCCTTCCTTGG
lgmn	F:	TGGACGATCCCGAGGATGG
	R:	GTGGATGATCTGGTAGGCGT
Ctsc	F:	CAACTGCACCTACCCTGATCT
	R:	TAAAATGCCCGGAATTGCCCA
Ptbp1	F:	CACACCCCAAAGCCTCTTTAT

	R:	ATCTGCACAAGTGCGTTCTCC
Ctsz	F:	GGCCAGACTTGCTACCATCC
	R:	ACACCGTTCACATTTCTCCAG
Otub1	F:	GTAGCGACTCCGAAGGTGTT
	R:	ACCAGAGGATTCTGCACAGC
Cxcl1	F:	CTGGGATTCACCTCAAGAACATC
	R:	CAGGGTCAAGGCAAGCCTC
Cxcl2	F:	CCAACCACCAGGCTACAGG
	R:	GCGTCACACTCAAGCTCTG
Ccl2	F:	TTAAAAACCTGGATCGGAACCAA
	R:	GCATTAGCTTCAGATTTACGGGT
Ccl7	F:	GCTGCTTTCAGCATCCAAGTG
	R:	CCAGGGACACCGACTACTG
Gss	F:	CAAAGCAGGCCATAGACAGGG
	R:	AAAAGCGTGAATGGGGCATAC
Glrx2	F:	ATCGTCGTTTTGGGGGAAGTC
	R:	GGAACAGTAAGAGCAGGATGTTT
Txn	F:	CATGCCGACCTTCCAGTTTTA
	R:	TTTCCTTGTTAGCACCGGAGA
Gsto1	F:	TAATTTGACCTTCTGGCCTA
	R:	GAAAGTATGGGGAAATCACA
Fiaf	F:	CATCCTGGGACGAGATGAACT
	R:	TGACAAGCGTTACCACAGGC
Adrp	F:	GACCTTGTGTCCTCCGCTTAT
	R:	CAACCGCAATTTGTGGCTC
Cpt1a	F:	CTCCGCCTGAGCCATGAAG
	R:	CACCAGTGATGATGCCATTCT
