# Supplementary material for: Oxidative stress-induced FABP5 S-glutathionylation protects against acute lung injury by suppressing inflammation in macrophages
Source: Nat Commun. 2021 Dec 7;12:7094. doi: 10.1038/s41467-021-27428-9 (PMC8651733; doi:10.1038/s41467-021-27428-9)
Supplement: Supplementary file 3 — Description of Additional Supplementary Files [file 41467_2021_27428_MOESM3_ESM.docx]

**Description of Additional Supplementary Files**

File Name: Supplementary Data 1

Description: Identification of specific SSG-modified sites by mass spectrometry.

File Name: Supplementary Data 2

Description: Functional classification of the proteome. Classification based on Gene Ontology (GO) categories.

.
