# Supplementary material for: Oxidative stress-induced FABP5 S-glutathionylation protects against acute lung injury by suppressing inflammation in macrophages
Source: Nat Commun. 2021 Dec 7;12:7094. doi: 10.1038/s41467-021-27428-9 (PMC8651733; doi:10.1038/s41467-021-27428-9)
Supplement: Supplementary file 6 — Reporting Summary [file 41467_2021_27428_MOESM6_ESM.pdf]

nature research

Corresponding author(s): Xue Zhang

Last updated by author(s): Nov 7, 2021

Reporting Summary

Nature Research wishes to improve the reproducibility of the work that we publish. This form provides structure for consistency and transparency in reporting. For further information on Nature Research policies, see our Editorial Policies and the Editorial Policy Checklist.

Statistics

For all statistical analyses, confirm that the following items are present in the figure legend, table legend, main text, or Methods section.

n/a	Confirmed	
☐	☒	The exact sample size (*n*) for each experimental group/condition, given as a discrete number and unit of measurement
☐	☒	A statement on whether measurements were taken from distinct samples or whether the same sample was measured repeatedly
☐	☒	The statistical test(s) used AND whether they are one- or two-sided<br>*Only common tests should be described solely by name; describe more complex techniques in the Methods section.*
☒	☐	A description of all covariates tested
☒	☐	A description of any assumptions or corrections, such as tests of normality and adjustment for multiple comparisons
☐	☒	A full description of the statistical parameters including central tendency (e.g. means) or other basic estimates (e.g. regression coefficient) AND variation (e.g. standard deviation) or associated estimates of uncertainty (e.g. confidence intervals)
☐	☒	For null hypothesis testing, the test statistic (e.g. *F*, *t*, *r*) with confidence intervals, effect sizes, degrees of freedom and *P* value noted<br>*Give P values as exact values whenever suitable.*
☒	☐	For Bayesian analysis, information on the choice of priors and Markov chain Monte Carlo settings
☒	☐	For hierarchical and complex designs, identification of the appropriate level for tests and full reporting of outcomes
☒	☐	Estimates of effect sizes (e.g. Cohen's *d*, Pearson's *r*), indicating how they were calculated

Our web collection on statistics for biologists contains articles on many of the points above.

Software and code

Policy information about availability of computer code

Data collection	Olympus BX61 (Olympus), Olympus VS200 (Olympus), Nikon Eclipse Ci-S (Nikon), ACEA NovoCyte™(ACEA Biosciences), CFX96 Touch™ Real-Time PCR Detection System (Bio-Rad), Amaxa Nucleofector II (Amaxa), Promega GloMax 20/20 (Promega), SynergyMx M5 (Molecular Devices).
Data analysis	GraphPad Prism 8 (GraphPad Software), FlowJo™ v10 (Tree Star), Schrödinger pacage 2017-04 (Schrödinger), CovDock (Schrödinger), OlyVIA 3.1 (Olympus), PyMOL 2.3.0 (DeLano Scientific LLC), RStudio 1.3.1056, MaxQuant search engine v.1.5.2.8, ClustalX2, DESeq2 package (1.20.0), MaxQuant search engine (v.1.5.2.8), GeneDoc 2.7.

For manuscripts utilizing custom algorithms or software that are central to the research but not yet described in published literature, software must be made available to editors and reviewers. We strongly encourage code deposition in a community repository (e.g. GitHub). See the Nature Research guidelines for submitting code & software for further information.

Data

Policy information about availability of data

All manuscripts must include a data availability statement. This statement should provide the following information, where applicable:

- Accession codes, unique identifiers, or web links for publicly available datasets
- A list of figures that have associated raw data
- A description of any restrictions on data availability

The RNA-seq data generated in this study have been deposited in the NCBI gene expression Omnibus (GEO) under accession code GSE182238. The raw Mass Spectrum data generated in this study have been deposited in the PRIDE database under accession code PXD027965. Mass Spectrum data that supporting our findings have been deposited in Supplementary Table 1, 2. Structural models used in Fig. 6a, 6e, 6f were accessed from the Protein Data Bank under the accession code 4LKT. Structural models used in Fig. 6g were accessed from the Protein Data Bank under the accession codes 4AZM. The remaining data are available within

the Article, Supplementary Information or Source Data file. Source data are provided with this paper.

Field-specific reporting

Please select the one below that is the best fit for your research. If you are not sure, read the appropriate sections before making your selection.

☒ Life sciences ☐ Behavioural & social sciences ☐ Ecological, evolutionary & environmental sciences

For a reference copy of the document with all sections, see nature.com/documents/nr-reporting-summary-flat.pdf

Life sciences study design

All studies must disclose on these points even when the disclosure is negative.

Sample size	Sample size estimates has been performed on previous experience to obtain statistical significance and reproducibility. For in vitro experiments such as Western blot, qPCR, IHC, CO-IP , at least three samples were used per group for minimal statistics requirements. For in vivo studies, the sample size was determined to be sufficient to obtain the statistical difference between groups, and genotype. All experiments were repeated at least three times.
Data exclusions	No data was excluded.
Replication	All experiments were repeated independently at least three times with similar results. Many experiments were repeated independently by two lab members in the lab.
Randomization	All mice and tissue samples grouped randomly and blindly, except the genotype factors. Because we determined the genotype using genomic PCR before the experiments.
Blinding	All analysis were performed blind. The investigators were blinded to group allocation during data collection. Data analysis were performed by different investigators responsible for genotyping and analysis to avoid conscious and unconscious bias.

Behavioural & social sciences study design

All studies must disclose on these points even when the disclosure is negative.

Study description	*Briefly describe the study type including whether data are quantitative, qualitative, or mixed-methods (e.g. qualitative cross-sectional, quantitative experimental, mixed-methods case study).*
Research sample	*State the research sample (e.g. Harvard university undergraduates, villagers in rural India) and provide relevant demographic information (e.g. age, sex) and indicate whether the sample is representative. Provide a rationale for the study sample chosen. For studies involving existing datasets, please describe the dataset and source.*
Sampling strategy	*Describe the sampling procedure (e.g. random, snowball, stratified, convenience). Describe the statistical methods that were used to predetermine sample size OR if no sample-size calculation was performed, describe how sample sizes were chosen and provide a rationale for why these sample sizes are sufficient. For qualitative data, please indicate whether data saturation was considered, and what criteria were used to decide that no further sampling was needed.*
Data collection	*Provide details about the data collection procedure, including the instruments or devices used to record the data (e.g. pen and paper, computer, eye tracker, video or audio equipment) whether anyone was present besides the participant(s) and the researcher, and whether the researcher was blind to experimental condition and/or the study hypothesis during data collection.*
Timing	*Indicate the start and stop dates of data collection. If there is a gap between collection periods, state the dates for each sample cohort.*
Data exclusions	*If no data were excluded from the analyses, state so OR if data were excluded, provide the exact number of exclusions and the rationale behind them, indicating whether exclusion criteria were pre-established.*
Non-participation	*State how many participants dropped out/declined participation and the reason(s) given OR provide response rate OR state that no participants dropped out/declined participation.*
Randomization	*If participants were not allocated into experimental groups, state so OR describe how participants were allocated to groups, and if allocation was not random, describe how covariates were controlled.*

Ecological, evolutionary & environmental sciences study design

All studies must disclose on these points even when the disclosure is negative.

Study description	*Briefly describe the study. For quantitative data include treatment factors and interactions, design structure (e.g. factorial, nested, hierarchical), nature and number of experimental units and replicates.*
Research sample	*Describe the research sample (e.g. a group of tagged Passer domesticus, all Stenocereus thurberi within Organ Pipe Cactus National Monument), and provide a rationale for the sample choice. When relevant, describe the organism taxa, source, sex, age range and any manipulations. State what population the sample is meant to represent when applicable. For studies involving existing datasets, describe the data and its source.*
Sampling strategy	*Note the sampling procedure. Describe the statistical methods that were used to predetermine sample size OR if no sample-size calculation was performed, describe how sample sizes were chosen and provide a rationale for why these sample sizes are sufficient.*
Data collection	*Describe the data collection procedure, including who recorded the data and how.*
Timing and spatial scale	*Indicate the start and stop dates of data collection, noting the frequency and periodicity of sampling and providing a rationale for these choices. If there is a gap between collection periods, state the dates for each sample cohort. Specify the spatial scale from which the data are taken*
Data exclusions	*If no data were excluded from the analyses, state so OR if data were excluded, describe the exclusions and the rationale behind them, indicating whether exclusion criteria were pre-established.*
Reproducibility	*Describe the measures taken to verify the reproducibility of experimental findings. For each experiment, note whether any attempts to repeat the experiment failed OR state that all attempts to repeat the experiment were successful.*
Randomization	*Describe how samples/organisms/participants were allocated into groups. If allocation was not random, describe how covariates were controlled. If this is not relevant to your study, explain why.*
Blinding	*Describe the extent of blinding used during data acquisition and analysis. If blinding was not possible, describe why OR explain why blinding was not relevant to your study.*

Did the study involve field work? ☐ Yes ☐ No

Field work, collection and transport

Field conditions	*Describe the study conditions for field work, providing relevant parameters (e.g. temperature, rainfall).*
Location	*State the location of the sampling or experiment, providing relevant parameters (e.g. latitude and longitude, elevation, water depth).*
Access & import/export	*Describe the efforts you have made to access habitats and to collect and import/export your samples in a responsible manner and in compliance with local, national and international laws, noting any permits that were obtained (give the name of the issuing authority, the date of issue, and any identifying information).*
Disturbance	*Describe any disturbance caused by the study and how it was minimized.*

Reporting for specific materials, systems and methods

We require information from authors about some types of materials, experimental systems and methods used in many studies. Here, indicate whether each material, system or method listed is relevant to your study. If you are not sure if a list item applies to your research, read the appropriate section before selecting a response.

Materials & experimental systems

n/a	Involved in the study	
☐	☒	Antibodies
☐	☒	Eukaryotic cell lines
☒	☐	Palaeontology and archaeology
☐	☒	Animals and other organisms
☒	☐	Human research participants
☒	☐	Clinical data
☒	☐	Dual use research of concern

Methods

n/a	Involved in the study	
☒	☐	ChIP-seq
☐	☒	Flow cytometry
☒	☐	MRI-based neuroimaging

Antibodies

Antibodies used

Anti-Glutathione (Abcam, clone D8, Cat#ab19534, Lot#GR3247576-7, WB:1:1000, IP:1:50)
Anti-Glutaredoxin 1 (Abcam, Cat#45953, Lot#GR3223671-2, WB:1:250, IF:1:100)
Anti-mouse IgG, HRP-linked Antibody (Cell Signaling Technology, Cat#7076, Lot#34, WB:1:1000)
Anti-rabbit IgG, HRP-linked Antibody (Cell Signaling Technology, Cat#7074, Lot#28,WB:1:1000)
FABP5 Rabbit mAb (D1A7T,Cell Signaling Technology, Cat#39926,Lot#1, WB:1:1000)
FABP5 Polyclonal Antibody (Proteintech, Cat#12348-1-AP, Lot#00051428, WB:1:500, IF:1:100)
Lamin A/C Mouse mAb (4C11, Cell Signaling Technology, Cat#4777, WB:1:2000)
Lamin B1 Monoclonal Antibody (Proteintech, clone 3C10G12, Cat#66095-1-Ig , Lot#10004949, WB:1:1000)
β-actin (A2-F6, Huabio, Cat#M1210-2, Lot#HL0305, WB:1:2000)
Anti-Flag (Cell Signaling Technology, Cat#14793S, Lot#5, 1:30)
PPARβ/δ (Santa Cruz, Cat#sc-74517, Lot#12320, WB: 1:500)
streptavidin-conjugated Alexa Fluor™ 488 (Invitrogen, Cat#S32354, Lot#1719656, IF: 0.5 μg/mL)
Alexa Fluor™ 488 donkey anti-rabbit IgG (Invitrogen, Cat#A21206, Lot#2156521, IF:1:200)
Alexa Fluor™ 488 donkey anti-mouse IgG (Invitrogen, Cat#A21202, Lot#2090565, IF:1:200)
anti-IgG (Thermo Fisher Scientific, Cat#26157, CHIP:1:250, anti-IgG is a component of ChIP Kit)

Validation

https://www.abcam.com/glutathione-antibody-d8-ab19534.html
https://www.abcam.com/glutaredoxin-1-antibody-ab45953.html
https://www.cellsignal.cn/products/secondary-antibodies/anti-mouse-igg-hrp-linked-antibody/7076
https://www.cellsignal.cn/products/secondary-antibodies/anti-rabbit-igg-hrp-linked-antibody/7074
https://www.cellsignal.cn/products/primary-antibodies/fabp5-d1a7t-rabbit-mab/39926
http://www.ptgcn.com/products/FABP5-Antibody-12348-1-AP.htm
https://www.cellsignal.cn/products/primary-antibodies/lamin-a-c-4c11-mouse-mab/4777
http://www.ptgcn.com/products/LMNB1-Antibody-66095-1-Ig.htm
http://www.huabio.cn/product/Beta-actin-antibody-M1210-2
https://www.cellsignal.com/products/primary-antibodies/dykddddk-tag-d6w5b-rabbit-mab-binds-to-same-epitope-as-sigma-s-anti-flag-m2-antibody/14793?site-search-type=Products&N=4294956287&Ntt=14793s&fromPage=plp&_requestid=3335746
https://www.scbt.com/p/pparbeta-antibody-f-10
https://www.thermofisher.com/order/catalog/product/S32354#/S32354
https://www.thermofisher.com/cn/zh/antibody/product/Donkey-anti-Rabbit-IgG-H-L-Highly-Cross-Adsorbed-Secondary-Antibody-Polyclonal/A-21206
https://www.thermofisher.com/cn/zh/antibody/product/Donkey-anti-Mouse-IgG-H-L-Highly-Cross-Adsorbed-Secondary-Antibody-Polyclonal/A-21202
https://www.thermofisher.cn/order/catalog/product/26157?SID=srch-srp-26157

Antibodies were chosen based on the available literature. All antibodies were provided by the manufacturer with validation data and citations.

Eukaryotic cell lines

Policy information about cell lines

Cell line source(s)

Raw264.7 cells, COS-7 cells, MH-S cells, MLE-12 cells, HFL1 cells, A549 cells and EA.hy926 cells were obtained from ATCC.

Authentication

Identity of these cell lines were frequently checked by their morphological features.

Mycoplasma contamination

All cell lines were tested to be mycoplasma-negative by Mycoplasma Detection Kit.

Commonly misidentified lines
(See ICLAC register)

No commonly misidentified cell lines are used in this study.

Palaeontology and Archaeology

Specimen provenance

Provide provenance information for specimens and describe permits that were obtained for the work (including the name of the issuing authority, the date of issue, and any identifying information).

Specimen deposition

Indicate where the specimens have been deposited to permit free access by other researchers.

Dating methods

If new dates are provided, describe how they were obtained (e.g. collection, storage, sample pretreatment and measurement), where they were obtained (i.e. lab name), the calibration program and the protocol for quality assurance OR state that no new dates are provided.

☐ Tick this box to confirm that the raw and calibrated dates are available in the paper or in Supplementary Information.

Ethics oversight

Identify the organization(s) that approved or provided guidance on the study protocol, OR state that no ethical approval or guidance was required and explain why not.

Note that full information on the approval of the study protocol must also be provided in the manuscript.

Animals and other organisms

Policy information about studies involving animals; ARRIVE guidelines recommended for reporting animal research

Laboratory animals	LysM cre Grx1 fl/fl, LysM cre FABP5 fl/fl, Grx1 KO, FABP5 KO mice were used: Both male and female ranging from 8-10 weeks of age. In some experiments P7 and P14 mice were used. Mice were housed under specific pathogen-free conditions. All mice were cultured in suitable temperature and humidity environment and fed with sufficient water and food (25 ℃, suitable humidity (typically 50%), 12 hour dark/light cycle).
Wild animals	This study did not involve wild animals.
Field-collected samples	The study did not involve samples collected from the field.
Ethics oversight	All animal experiments were performed according to protocols approved by the Institutional Animal Care and Use Committee of Zhejiang University School of Medicine (ETHICS CODE: ZJU20200180).

Note that full information on the approval of the study protocol must also be provided in the manuscript.

Human research participants

Policy information about studies involving human research participants

Population characteristics	*Describe the covariate-relevant population characteristics of the human research participants (e.g. age, gender, genotypic information, past and current diagnosis and treatment categories). If you filled out the behavioural & social sciences study design questions and have nothing to add here, write "See above."*
Recruitment	*Describe how participants were recruited. Outline any potential self-selection bias or other biases that may be present and how these are likely to impact results.*
Ethics oversight	*Identify the organization(s) that approved the study protocol.*

Note that full information on the approval of the study protocol must also be provided in the manuscript.

Clinical data

Policy information about clinical studies

All manuscripts should comply with the ICMJEguidelines for publication of clinical research and a completedCONSORT checklist must be included with all submissions.

Clinical trial registration	*Provide the trial registration number from ClinicalTrials.gov or an equivalent agency.*
Study protocol	*Note where the full trial protocol can be accessed OR if not available, explain why.*
Data collection	*Describe the settings and locales of data collection, noting the time periods of recruitment and data collection.*
Outcomes	*Describe how you pre-defined primary and secondary outcome measures and how you assessed these measures.*

Dual use research of concern

Policy information about dual use research of concern

Hazards

Could the accidental, deliberate or reckless misuse of agents or technologies generated in the work, or the application of information presented in the manuscript, pose a threat to:

No	Yes	
☐	☐	Public health
☐	☐	National security
☐	☐	Crops and/or livestock
☐	☐	Ecosystems
☐	☐	Any other significant area

Experiments of concern

Does the work involve any of these experiments of concern:

No	Yes	
☐	☐	Demonstrate how to render a vaccine ineffective
☐	☐	Confer resistance to therapeutically useful antibiotics or antiviral agents
☐	☐	Enhance the virulence of a pathogen or render a nonpathogen virulent
☐	☐	Increase transmissibility of a pathogen
☐	☐	Alter the host range of a pathogen
☐	☐	Enable evasion of diagnostic/detection modalities
☐	☐	Enable the weaponization of a biological agent or toxin
☐	☐	Any other potentially harmful combination of experiments and agents

ChIP-seq

Data deposition

☐ Confirm that both raw and final processed data have been deposited in a public database such as GEO.

☐ Confirm that you have deposited or provided access to graph files (e.g. BED files) for the called peaks.

Data access links<br>*May remain private before publication.*	*For "Initial submission" or "Revised version" documents, provide reviewer access links. For your "Final submission" document, provide a link to the deposited data.*
Files in database submission	*Provide a list of all files available in the database submission.*
Genome browser session<br>(e.g. UCSC)	*Provide a link to an anonymized genome browser session for "Initial submission" and "Revised version" documents only, to enable peer review. Write "no longer applicable" for "Final submission" documents.*

Methodology

Replicates	*Describe the experimental replicates, specifying number, type and replicate agreement.*
Sequencing depth	*Describe the sequencing depth for each experiment, providing the total number of reads, uniquely mapped reads, length of reads and whether they were paired- or single-end.*
Antibodies	*Describe the antibodies used for the ChIP-seq experiments; as applicable, provide supplier name, catalog number, clone name, and lot number.*
Peak calling parameters	*Specify the command line program and parameters used for read mapping and peak calling, including the ChIP, control and index files used.*
Data quality	*Describe the methods used to ensure data quality in full detail, including how many peaks are at FDR 5% and above 5-fold enrichment.*
Software	*Describe the software used to collect and analyze the ChIP-seq data. For custom code that has been deposited into a community repository, provide accession details.*

Flow Cytometry

Plots

Confirm that:

☒ The axis labels state the marker and fluorochrome used (e.g. CD4-FITC).

☒ The axis scales are clearly visible. Include numbers along axes only for bottom left plot of group (a 'group' is an analysis of identical markers).

☒ All plots are contour plots with outliers or pseudocolor plots.

☒ A numerical value for number of cells or percentage (with statistics) is provided.

Methodology

Sample preparation	Intracellular ROS levels were assessed using a 2′,7′-dichlorofluorescein diacetate (DCFH-DA) fluorescent probe (Sigma, D6683), using a final concentration of 1 mM, and analyzed on an ACEA NovoCyte Flow Cytometer.
Instrument	ACEA NovoCyte™(ACEA Biosciences) was used for data collection.

Software	FlowJo™ v10 (Tree Star) was used to analyze the data.
Cell population abundance	No sorting was used in this manuscript.
Gating strategy	First, cells were gated on the FSC/SSC plots, then single cells were gated on the FSC-H/FSC-A plots. Interested cells were stained with DCFH-DA fluorescent probe (Sigma, D6683). Gating was determined by blank and single color-staining.

☒ Tick this box to confirm that a figure exemplifying the gating strategy is provided in the Supplementary Information.

Magnetic resonance imaging

Experimental design

Design type	*Indicate task or resting state; event-related or block design.*
Design specifications	*Specify the number of blocks, trials or experimental units per session and/or subject, and specify the length of each trial or block (if trials are blocked) and interval between trials.*
Behavioral performance measures	*State number and/or type of variables recorded (e.g. correct button press, response time) and what statistics were used to establish that the subjects were performing the task as expected (e.g. mean, range, and/or standard deviation across subjects).*

Acquisition

Imaging type(s)	*Specify: functional, structural, diffusion, perfusion.*
Field strength	*Specify in Tesla*
Sequence & imaging parameters	*Specify the pulse sequence type (gradient echo, spin echo, etc.), imaging type (EPI, spiral, etc.), field of view, matrix size, slice thickness, orientation and TE/TR/flip angle.*
Area of acquisition	*State whether a whole brain scan was used OR define the area of acquisition, describing how the region was determined.*

Diffusion MRI ☐ Used ☐ Not used

Preprocessing

Preprocessing software	*Provide detail on software version and revision number and on specific parameters (model/functions, brain extraction, segmentation, smoothing kernel size, etc.).*
Normalization	*If data were normalized/standardized, describe the approach(es): specify linear or non-linear and define image types used for transformation OR indicate that data were not normalized and explain rationale for lack of normalization.*
Normalization template	*Describe the template used for normalization/transformation, specifying subject space or group standardized space (e.g. original Talairach, MNI305, ICBM152) OR indicate that the data were not normalized.*
Noise and artifact removal	*Describe your procedure(s) for artifact and structured noise removal, specifying motion parameters, tissue signals and physiological signals (heart rate, respiration).*
Volume censoring	*Define your software and/or method and criteria for volume censoring, and state the extent of such censoring.*

Statistical modeling & inference

Model type and settings	*Specify type (mass univariate, multivariate, RSA, predictive, etc.) and describe essential details of the model at the first and second levels (e.g. fixed, random or mixed effects; drift or auto-correlation).*
Effect(s) tested	*Define precise effect in terms of the task or stimulus conditions instead of psychological concepts and indicate whether ANOVA or factorial designs were used.*

Specify type of analysis: ☐ Whole brain ☐ ROI-based ☐ Both

Statistic type for inference (See Eklund et al. 2016)	*Specify voxel-wise or cluster-wise and report all relevant parameters for cluster-wise methods.*
Correction	*Describe the type of correction and how it is obtained for multiple comparisons (e.g. FWE, FDR, permutation or Monte Carlo).*

Models & analysis

n/a	Involved in the study
☐	☐ Functional and/or effective connectivity
☐	☐ Graph analysis
☐	☐ Multivariate modeling or predictive analysis

Functional and/or effective connectivity	*Report the measures of dependence used and the model details (e.g. Pearson correlation, partial correlation, mutual information).*
Graph analysis	*Report the dependent variable and connectivity measure, specifying weighted graph or binarized graph, subject- or group-level, and the global and/or node summaries used (e.g. clustering coefficient, efficiency, etc.).*
Multivariate modeling and predictive analysis	*Specify independent variables, features extraction and dimension reduction, model, training and evaluation metrics.*
